# Adaptive two-stage bioequivalence trials with early stopping and sample size re-estimation

**DOI:** 10.1186/1745-6215-16-S2-P218

**Published:** 2015-11-16

**Authors:** Franz Koenig, Martin Wolfsegger, Thomas Jaki, Helmut Schütz, Gernot Wassmer

**Affiliations:** 1 Lancaster University, Lancaster, UK; 2 Medical University Vienna, Vienna, Austria; 3 Baxter Innovations GmbH, Vienna, Austria; 4 BEBAC - Consultancy Services for Bioequivalence and Bioavailability Studies, Vienna, Austria; 5 University Köln, Köln, Germany

## 

Bioequivalence between two products has to be demonstrated as an essential part of the generic approval process (new formulation vs. innovator product), bridging an innovator's product from the formulation used in clinical phase III to the marketed formulation, or in case of major variations of an approved product. The most common design of bioequivalence studies is a two-sequence two-period two-treatment crossover design, where inclusion of 90% confidence intervals of pharmacokinetic metrics in a pre-defined acceptance range has to be shown. Alternatively, bioequivalence can be established by using Two One-Sided Tests (TOST) each at an alpha level of 5%. However, this fixed sample approach offers no flexibility if in the planning phase parameters were misspecified.

We propose a two-stage adaptive design based using combination tests to combine stagewise p-values. This will strictly control the type I error rate in case data-driven design modification have to be performed at an interim analysis. We derive 90%-repeated confidence intervals for the adaptive TOST approach. We investigate different sample size reassessment strategies using conditional power arguments. We discuss how futility stopping can be sensible implemented. The operating characteristics will be assessed by clinical trial simulations.

Furthermore, the proposed adaptive design would allow to switch from a classical two-period design to a more complex replicate design if it turns out the reference product is highly variable and the within subject-variability has to be determined as well. Another application of the proposed method would be for establishing biosimilarity of two products.

